# Telomere repeat–binding factor 2 binds extensively to extra-telomeric G-quadruplexes and regulates the epigenetic status of several gene promoters

**DOI:** 10.1074/jbc.RA119.008687

**Published:** 2019-10-01

**Authors:** Ananda Kishore Mukherjee, Shalu Sharma, Sulochana Bagri, Rintu Kutum, Pankaj Kumar, Asgar Hussain, Prateek Singh, Dhurjhoti Saha, Anirban Kar, Debasis Dash, Shantanu Chowdhury

**Affiliations:** ‡ Integrative and Functional Biology Unit, Council of Scientific and Industrial Research (CSIR)-Institute of Genomics and Integrative Biology (IGIB), Mathura Road, New Delhi 110025, India; § Academy of Scientific and Innovative Research (AcSIR), Council of Scientific and Industrial Research (CSIR)-Institute of Genomics and Integrative Biology (IGIB), Mathura Road, New Delhi 110025, India; ¶ G.N.R. Knowledge Centre for Genome Informatics, Council of Scientific and Industrial Research (CSIR)-Institute of Genomics and Integrative Biology (IGIB), Mathura Road, New Delhi 110025, India; ‖ CSIR Ayurgenomics Unit-TRISUTRA, Council of Scientific and Industrial Research (CSIR)-Institute of Genomics and Integrative Biology (IGIB), Mathura Road, New Delhi 110025, India

**Keywords:** DNA transcription, DNA-binding protein, G-quadruplex, epigenetics, telomere, extra-telomeric, genome-wide, telomere repeat-binding factor 2, TRF2, gene regulation, shelterin complex, DNA secondary structure, histone mark

## Abstract

The role of the telomere repeat-binding factor 2 (TRF2) in telomere maintenance is well-established. However, recent findings suggest that TRF2 also functions outside telomeres, but relatively little is known about this function. Herein, using genome-wide ChIP-Seq assays of TRF2-bound chromatin from HT1080 fibrosarcoma cells, we identified thousands of TRF2-binding sites within the extra-telomeric genome. In light of this observation, we asked how TRF2 occupancy is organized within the genome. Interestingly, we found that extra-telomeric TRF2 sites throughout the genome are enriched in potential G-quadruplex–forming DNA sequences. Furthermore, we validated TRF2 occupancy at several promoter G-quadruplex motifs, which did adopt quadruplex forms in solution. TRF2 binding altered expression and the epigenetic state of several target promoters, indicated by histone modifications resulting in transcriptional repression of eight of nine genes investigated here. Furthermore, TRF2 occupancy and target gene expression were also sensitive to the well-known intracellular G-quadruplex–binding ligand 360A. Together, these results reveal an extensive genome-wide association of TRF2 outside telomeres and that it regulates gene expression in a G-quadruplex–dependent fashion.

## Introduction

TRF2, as part of the shelterin complex, confers stability to telomeres (1–5). Telomere ends can be vulnerable to double-strand breaks and therefore need protection from the cellular double-stranded break repair machinery (6–9). TRF2 plays a critical role in how telomere ends evade detection as damaged DNA by blocking the ATM^5^ and ATR kinases from triggering DNA damage response (10). For this and other telomere-related functions (10–12), TRF2 has been studied as a telomeric factor (13, 14), where it is known to associate with double-stranded telomeric DNA as a homodimer from dimerization of the C-terminal MYB domains of TRF2 (10, 11). Interestingly, emerging findings implicate TRF2 in functions outside telomeres.

Extra-telomeric TRF2-binding sites were detected in two independent studies (15, 16). These support extra-telomeric TRF2 functions, including DNA repair (17) and recently observed transcriptional regulation (18–20). However, underlying mechanisms remain to be understood. For instance, whether and how TRF2 engages transcriptional co-activators (or repressors) and/or cognate DNA sequence motif(s) is not clear. Interestingly, a truncated form of TRF2 was noted to associate with the DNA secondary structure G-quadruplex (G4) motif formed in solution by telomeric TTAGGG repeats (21). TRF2-G4 interaction was also recently reported for the PCGF3 promoter G4 motif (20).

The core structure of the G4 motif comprises stacked planar tetrads of guanine residues stabilized by Hoogsteen base-pairing (22–26). Related lines of evidence support the biological role of G4 motifs (27, 28). For example, sequences with the potential to adopt the G4 motif are enriched and conserved in promoters from bacteria to higher eukaryotes (29–32); the role of the G4 motif in regulation of gene expression, replication, recombination, and telomere maintenance has been studied (33–36), and interaction of regulatory factors like NM23H2, MAZ/PARP-1, XPB/XPD, and the helicase Pif1 in yeast with potential G4-forming sequences was reported (37–41). Furthermore, the *in vivo* presence of G4 motifs was found in ciliate telomeres (42) and recently within interstitial/telomeric regions in human cells using G4 antibodies (43–45).

Previous TRF2 ChIP-Seq studies reported fewer than 200 sites genome-wide (15, 16). Because of the low read counts within peaks (much below the ENCODE recommendation of >1% of total reads (46); see below) and the low number of genomic sites (relative to most ChIP-Seq findings) reported in the two earlier papers, we first revisited the extent of TRF2 binding across the genome. Second, we considered our recent findings showing TRF2 occupancy at several promoters spread across the genome (47), where, interestingly, TRF2 binding was noted within G-rich sequences in many cases. Moreover, the TRF2 binding resulted in epigenetic and gene expression changes that were sensitive to telomere length. Prompted by these findings, herein we questioned whether the mode of TRF2 binding was G4 motif–dependent. We found >20,000 TRF2 sites genome-wide from replicate ChIP-Seq experiments (where ∼11% of reads were within peaks). We also observed a strong association between TRF2 binding and potential G4 (PG4) motif-forming sites throughout the genome. Further experiments revealed TRF2 occupancy, expression, and epigenetic state of the target promoter to be dependent on the TRF2–G4 motif interaction.

## Results

### Thousands of extra-telomeric TRF2-binding sites across the genome

We performed ChIP-Seq for endogenous TRF2 in HT1080 fibrosarcoma cells (see “Experimental procedures” for details of protocol and antibody). Of roughly 26.4 million reads, ∼15.8 million reads (60%) aligned to the human genome in each replicate. We noted that ∼3.6 million reads (23%) represented telomeric sequences (reads with (TTAGGG)_2_ repeats), as expected for a telomere-binding factor. To check the overall distribution of the aligned reads, the entire genome was divided into 50-bp bins; significant enrichment of TRF2 reads over input was found in 2.79% of the bins, suggesting selective distribution of TRF2 occupancy at a genome-wide level. Interestingly, we found more reads mapped to interstitial regions of the genome compared with the sub-telomeric regions (up to 0.5 Mb from chromosome termini; Table 1).

**Table 1:** **ChIP-Seq reads in sub-telomeric and interstitial regions**

	Average*^a^* number of reads mapping to sub-telomeric*^b^* region	Average*^a^* number of reads mapping to interstitial region
Chr 1	2933	236,762.5
Chr 2	1366.5	235,044
Chr 3	1761	189,761
Chr 4	2721.5	179,752
Chr 5	4848	175,975
Chr 6	1172	155,155.5
Chr 7	1469.5	170,633.5
Chr 8	1312	138,489
Chr 9	1915	128,188.5
Chr 10	2007.5	147,970
Chr 11	1756.5	132,698
Chr 12	2612.5	135,934
Chr 13	365.5	86,333
Chr 14	367.5	87,953.5
Chr 15	1054.5	82,229
Chr 16	1628	96,387.5
Chr 17	1093	91,077.5
Chr 18	3326	67,039.5
Chr 19	982.5	78,062
Chr 20	1759	59,221.5
Chr 21	715	40,403.5
Chr 22	560	39,900.5
Chr X	2441	151,597.5
Chr Y	10,821.5	44,276.5

*^a^* Average from two replicate ChIP-Seq experiments.

*^b^* Sub-telomeric considered as regions up to 0.5 Mb away from chromosomal termini.

**Figure 1.**
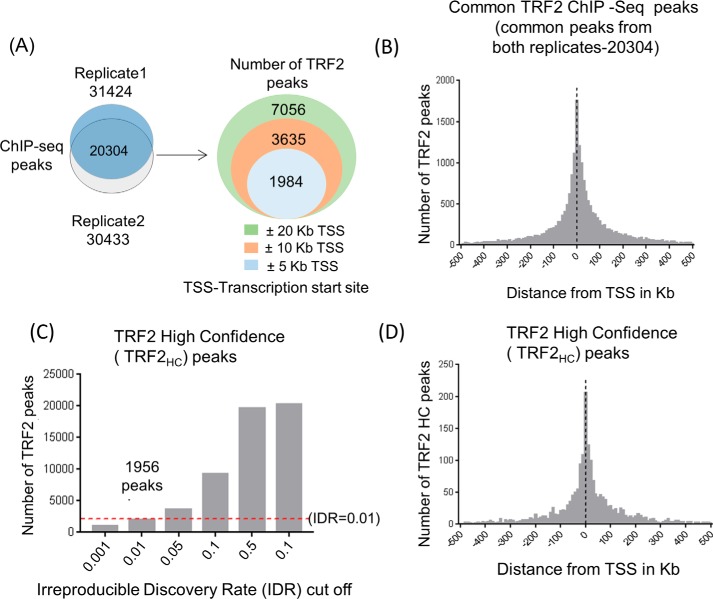
**Thousands of extra-telomeric TRF2 binding sites found across the genome.** *A*, extra-telomeric TRF2 peaks found in HT1080 cells following TRF2 ChIP-Seq: 20304 TRF2 peaks were common between two independent experiments. *B*, distribution of common TRF2 peaks around TSSs. Distance from the TSS is shown in kb. *C*, replicate consistency plot generated using irreproducible discovery rate analysis; 1956 peaks found at ≤0.01 are *marked. D*, distribution of 1956 TRF2 peaks with IDR 0.01 (TRF2_HC_ peaks) around the TSS. Distance from the TSS is shown in kb.

Of the aligned reads, 2,999,796 (18.9%) and 3,041,641 (19.1%) contributed to 31,424 and 30,433 TRF2 peaks, respectively, in two replicate experiments. Of these, 20,304 TRF2 peaks were common between the two ChIP-Seq replicates (supporting information). Of the common peaks, 7056, 3635, and 1984 sites mapped within 20, 10, or 5 kb of the transcription start sites (TSS), respectively (Fig. 1*A*). Moreover, we noted that the number of TRF2 peaks significantly increased in regions near TSS across the genome (*p* < 0.05; Fisher's exact test; Fig. 1*B*).

For further analyses, we used irreproducible discovery rate (IDR), a widely accepted measure of consistency between replicates that utilize high-throughput techniques (46). Of 20,304 TRF2 peaks (common to both replicates), 1956 had IDR ≤ 0.01 (the ENCODE recommended cut-off for peaks detected with high confidence (46)) and were designated as TRF2-high-confidence (TRF2_HC_) peaks (Fig. 1*C* and supporting information). TRF2_HC_ peaks were also substantially enriched near promoter regions (Fig. 1*D*). TRF2_HC_ peaks were nonrandomly distributed across all chromosomes, with overall 23.6% of the peaks within 10 kb of TSS; 6.55, 0.56, and 69.29% were within 5 kb downstream of transcription end sites, coding exons and introns, respectively (Fig. S1), and 465 of the 1956 TRF2 peaks had at least one interstitial TTAGGG sequence.

### Comparative analysis of TRF2 peaks reported here with earlier data

In an earlier study, 77 TRF2 peaks were reported in HTC75 cells (16). Of the 77 peaks, peak coordinates for 50 TRF2 sites near genes were reported. Fourteen of these overlapped with TRF2 peaks found in our study. In a second study, 183 TRF2 peaks were reported in BJ fibroblast cells using monoclonal and polyclonal antibodies; 72 of the 183 peaks were common to polyclonal/monoclonal experiments (15). Of these 72, 68 peak coordinates common to TRF1 or TRF2 were reported (15). Intriguingly, none of the 68 sites overlapped with the 50 TRF2 sites reported earlier (16) or TRF2 peaks found in our study.

On re-analysis of earlier data from BJ fibroblast cells (15) with the pipeline used by us here (MACS 1.4), we found 283 or 144 TRF2 peaks (from data reported using monoclonal or polyclonal anti-TRF2 antibody, respectively; supporting information). Of these, 37 and 26 peaks in monoclonal or polyclonal antibody, respectively, overlapped with our data (supporting information).We compared the peak calling results based on ChIP-Seq standards recommended by ENCODE, which prescribes that the fraction of reads in peaks (FRiP value) should be higher than 1% (48). FRiP values for the monoclonal and polyclonal antibody experiments were 0.23 and 0.19%, respectively (15). Compared with this, in our ChIP-Seq data, up to ∼11% of reads were within the identified peaks. We believe that the notably higher (>40-fold) number of aligned reads within peaks (compared with the earlier report) is the most likely reason for the substantially increased number of TRF2 peaks reported here. Possible reasons, in addition to the low number of aligned reads, could be the different cell lines used in the studies. Raw data for re-analyses were not available for the second study (16).

### Extra-telomeric TRF2 peaks harbor G-quadruplex motifs genome-wide

**Figure 2.**
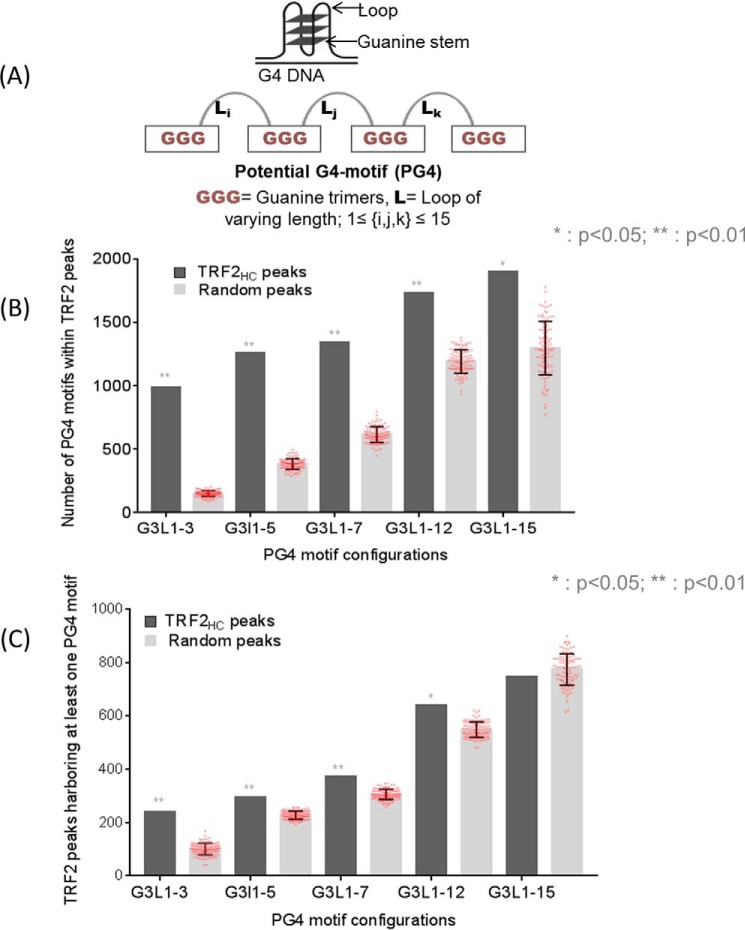
**TRF2 peaks harbor G-quadruplex motifs genome-wide.** *A*, *schematic representation* of a G4 motif; sequence pattern with loop/stem and PG4 motif formed by a tetrad of guanine trimers interspersed with loops that can vary in length. *B* and *C*, PG4 motifs and TRF2 peaks significantly overlap. High-confidence TRF2-binding sites (TRF2_HC_ peaks) determined by ChIP-Seq in HT1080 cells were significantly enriched in PG4-motif sequences (*B*), and conversely, PG4-motif sequences were enriched within TRF2_HC_ peaks (*C*). Nonoverlapping PG4 motifs were considered for analysis; for control analysis, 100 regions of identical length for each TRF2 peak were taken. *, *p* < 0.05; **, *p* < 0.01 (Fisher's exact test). *Error bars*, S.D.

Interaction of TRF2 with G4 motifs in solution was reported earlier (21). Herein, we sought to check whether TRF2 ChIP-Seq peaks were associated with potential G4 (PG4)-forming sequences. For this, we used the frequently studied PG4 configuration(s) comprising four runs of three guanines (G3)_4_ with three intervening loops of up to 15 bases (as shown in Fig. 2*A*).

For each TRF2 peak, 100 regions of identical length were randomly selected from across the genome as control regions to analyze significance. The number of PG4 motifs within 1956 TRF2_HC_ peaks (or corresponding control regions) was mapped; this revealed PG4 motifs to be enriched within TRF2_HC_ peaks for different G4 loop length configurations (*p* < 0.05, Fisher's exact test; Fig. 2*B*). We next checked whether the converse was true (*i.e.* if the number of TRF2 peaks harboring at least one PG4 motif was significant). TRF2_HC_ peaks harboring one or more different PG4 configurations were significantly enriched compared with random peaks in all cases except the most relaxed G4 conformation, G3L1-15 (*p* < 0.05, Fisher's exact test; Fig. 2*C*).

### TRF2 interacts with promoter G4 motifs

**Figure 3.**
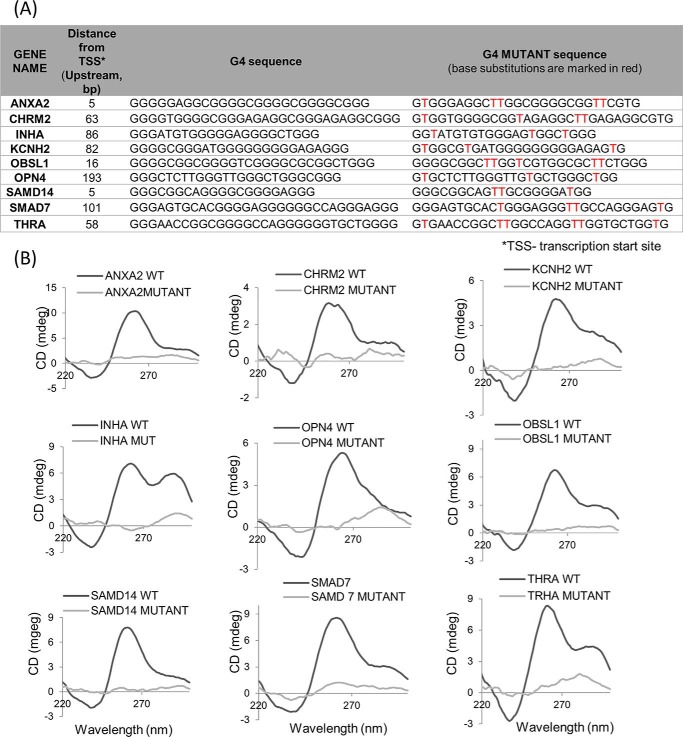
**Sequences within TRF2 peaks form G-quadruplex motifs.** *A*, *table* showing the location, distance from the TSS, and G4 motifs (WT and respective G4 mutant sequences of promoter PG4 motifs that overlap in TRF2 high-confidence peaks). *B*, CD plots of PG4-motif sequences shown in the above *table*; base-substituted mutant sequences that would not adopt G4 motifs were used as controls.

To test interaction of TRF2 with G4 motifs, we focused on nine promoters that had at least one PG4 motif within 500 bp of TSS and overlapped with the TRF2_HC_ peak (Fig. 3*A*). First, we checked whether the identified PG4 sequence adopted the G4 structure motif in solution using CD measurements; specific substitutions in critical guanine bases required for the G4 structure formation were made as negative control (Fig. 3*A*). CD plots indicating the formation of parallel G4 motifs (positive peak at 260 nm and a negative peak at 240 nm) was observed for all of the nine sequences tested, whereas negative control sequences in all cases did not show peaks for the G4 motif (Fig. 3*B*). Oligonucleotides corresponding to the genes *INHA* and *THRA* gave peaks at 290 nm in addition to the 240-nm peak, suggesting formation of mixed parallel/antiparallel G4 motifs.

Interaction of purified recombinant TRF2 with G4 motifs formed by sequences from the *p21* and *PCGF3* promoters (19, 20) and the telomeric G4 motif (20) in solution has been reported. Here, we tested this using PG4 sequences from two representative promoters: *SAMD14* and *CHRM2*. ELISAs with purified recombinant TRF2 and biotin-labeled oligonucleotides for *SAMD14* and *CHRM2* showed higher binding affinity with G4 motifs compared with corresponding negative controls (mutated or the flanking sequences; Fig. S2*A*).

In addition, for further confirmation of TRF2 binding with G4 motifs, we performed G4-fluorescent intercalator displacement (FID) assays using the G4 intercalator ligand thymidine orange (TO) as reported earlier and briefly described under “Experimental procedures” (49–51). TRF2 association with G4 motifs was observed from the measurement of TO displacement potential. DC_50_ values were indicative of TRF2 affinity for the respective G4 motifs in solution; interaction with human albumin instead of TRF2 was used as a negative control (Fig. S2*B*). Together, these showed that PG4 sequences identified within promoters formed G4 motifs and interacted with TRF2 in solution.

### TRF2 promoter occupancy mediates transcription regulation of the target gene

**Figure 4.**
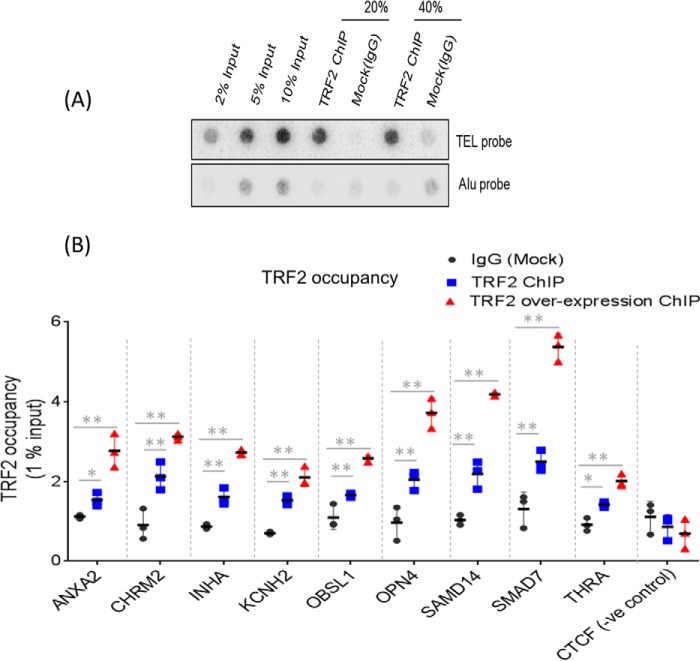
**TRF2 occupancy on G4-motif sites on gene promoters within cells.** *A*, telomeric enrichment was tested for TRF2 ChIP samples performed in HT1080 cells. Telomeric signal was normalized to signal from ALU probe. *B*, TRF2 occupancy at gene promoter sites in HT1080 cells (endogenous TRF2 and TRF2-overexpressed) quantified using ChIP-qRT PCR. TRF2 ChIP/IgG (mock) enrichment was normalized to 1% input; *CTCF* promoter (which does not harbor a TRF2 peak) was used as negative control; *error bars* correspond to S.D., and statistical significance was calculated by paired *t* test (*, *p* < 0.05; **, *p* < 0.01). For the TRF2-overexpressed condition (in *red*), all *p* values were <0.01.

Following this, we checked for intracellular TRF2 occupancy at sites overlapping the PG4 sequences within the nine promoters. Telomeric enrichment of TRF2 was confirmed using ChIP followed by Southern hybridization using telomere-specific probes (dot blots; Fig. 4*A*).

ChIP-PCR revealed TRF2 occupancy to be significant in all of the nine promoters (Fig. 4*B*); the *CTCF* promoter was used as a negative control (see “Experimental procedures”). We further noted that the enrichment of TRF2 on gene promoters increased when TRF2 was overexpressed for the majority of the target sites (Fig. 4*B* and Fig. 3*A*).

**Figure 5.**
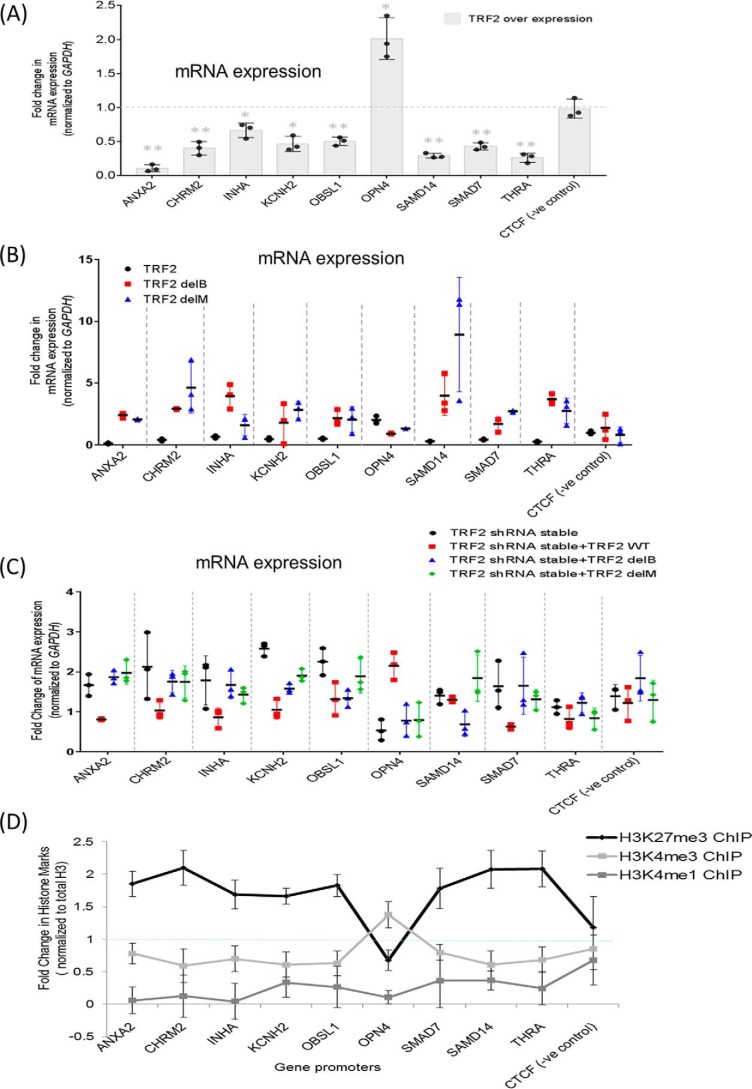
**TRF2-dependent transcriptional outcomes.** *A*, gene expression of target genes measured by qRT-PCR in untransfected and TRF2-overexpressed conditions in HT1080 cells. *GAPDH* expression was used for normalization; *error bars* correspond to S.D., and statistical significance was calculated by paired *t* test (*, *p* < 0.05; **, *p* < 0.01). *B*, effect of TRF2 domain mutations on expression of target genes. HT1080 cells were transfected with TRF2 WT or domain deletion mutants delB and delM, and expression was compared with control untransfected cells. *Error bars*, S.D. for three independent experiments. *C*, effect of TRF2 domain mutations on expression of target genes in TRF2-silenced background. HT1080 cells with stable TRF2 silencing were transfected with TRF2 WT or domain deletion mutants delB and delM, and expression was compared with SCR control and TRF2 shRNA–transfected cells. *Error bars*, S.D. for three independent experiments. *D*, H3K27me3 (suppression mark) and activation marks H3K4me3 and H3K4me1 enrichment (normalized to total H3) was tested within promoters in HT1080 cells following TRF2 overexpression relative to untransfected cells. *Error bars*, S.D. from two independent experiments.

Following this, we asked whether TRF2 occupancy resulted in altered mRNA expression of the nine target genes. In all of the nine cases, we found that TRF2 overexpression resulted in significantly altered mRNA expression of the corresponding gene (Fig. 5*A*); *CTCF*, with no TRF2 peak in its promoter, was used as a negative control gene. Interestingly, all genes, except *OPN4*, were transcriptionally repressed by TRF2. Moreover, further increase in TRF2 overexpression did not affect the overall transcriptional outcome (Fig. S3*B*).

To test the role of TRF2 DNA-binding domains in transcription, we expressed either the full-length TRF2, TRF2-delM (MYB domain deleted), or TRF2-delB (basic domain deleted) and checked expression of the target genes. Overexpression of TRF2 and the mutants was confirmed by Western blotting for DDK tag (Fig. 4*A*). Both TRF2-delB and TRF2-delM overexpression resulted in the loss of TRF2-induced transcription (repression/activation) of the genes (Fig. 5*B*). Furthermore, experiments were repeated in cells lacking endogenous TRF2. Following stable shRNA-mediated knockdown of TRF2, we overexpressed the WT or mutant forms (TRF2-delB and TRF2-delM) (Fig. S4*B*). Whereas the full-length WT TRF2 rescued the effect of TRF2 loss, expression of either TRF2-delB or TRF2-delM was not able to rescue TRF2-mediated transcriptional effects (*i.e.* resuppress the genes in most cases, or activate in case of OPN4) (Fig. 5*C*).

### TRF2 occupancy results in promoter histone modifications

In two earlier studies (19, 47), promoter TRF2 binding was found to result in histone modifications. Here, for the nine promoters, we tested whether this was consistent with altered target gene expression on TRF2 overexpression. For all genes, except *OPN4*, histone activation marks (H3K4me1/H3K4me3) were lower and/or the suppressor mark H3K27me3 increased at the respective promoter (Fig. 5*D*). In the case of OPN4, histone activation mark H3K4me3 increased. Together, this was consistent with TRF2-mediated expression observed for the nine genes.

### TRF2 occupancy at gene promoters was excluded in presence of the G4-binding ligand 360A

Following this, we sought to understand whether TRF2-mediated transcription regulation was G4-dependent. We reasoned that, in the case where TRF2 occupancy was G4-dependent, this was likely to be affected in the presence of G4-binding ligands. Therefore, the promoter occupancy of TRF2 at all of the nine promoter sites was checked following TRF2 overexpression in the presence or absence of the well-established intracellular G4-binding ligand 360A (52). The first report on 360A showed binding to a parallel G4 structure in the c-*MYC* promoter (53). Further, it was noted that 360A binds to telomeric G4s of either the hybrid or anti-parallel type (52) and also parallel G4 motifs from the *TP53* locus (54) and the *p21* promoter (19).

**Figure 6.**
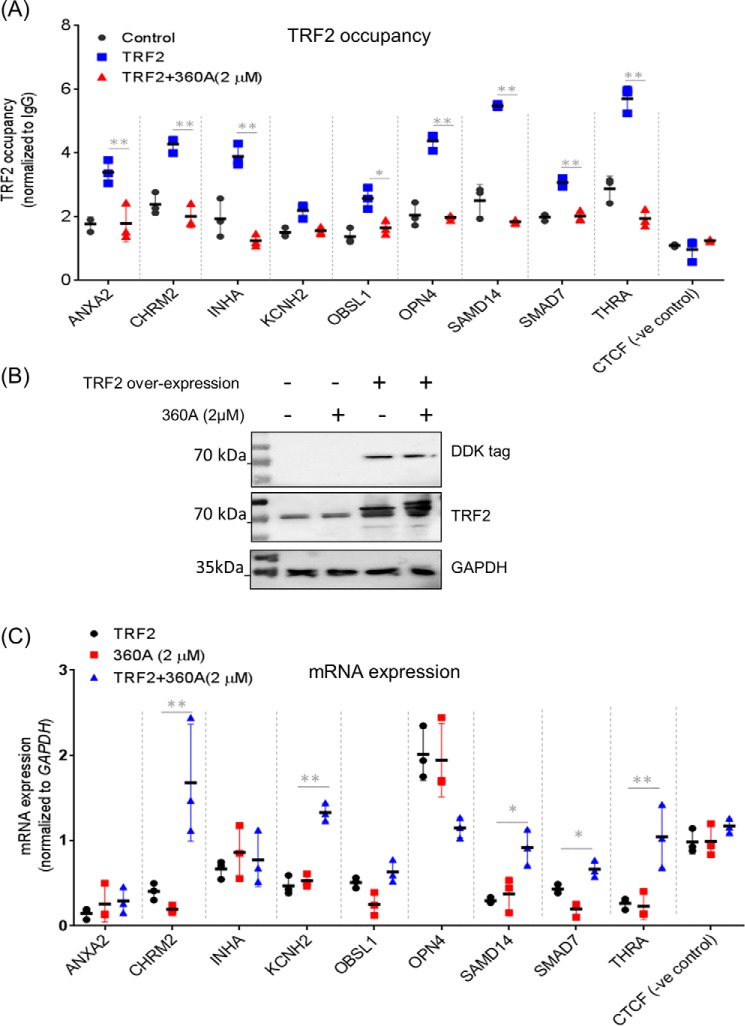
**TRF2 occupancy at gene promoters is sensitive to intracellular G-quadruplex–binding ligand 360A.** *A*, TRF2 occupancy at gene promoter sites was checked by ChIP qRT-PCR in HT1080 cells following overexpression of TRF2 in the presence or absence of 360A. *Error bars*, S.D. from three independent experiments; *CTCF* promoter was used as a negative control. *B*, TRF2 level was checked by Western blotting in the presence of the ligand 360A in both untransfected and TRF2 transient overexpression conditions. Overexpression was also confirmed by probing for DDK tag. GAPDH was used as loading control. *C*, expression of target genes was analyzed by qRT-PCR in HT1080 cells following overexpression of TRF2 in the presence or absence of 360A. *GAPDH* expression was used for normalization; *error bars*, S.D. from three independent experiments. Statistical significance was calculated by paired *t* test (*, *p* < 0.05; **, *p* < 0.01).

TRF2 occupancy at most of the gene promoters was reduced significantly in the presence of 360A (Fig. 6*A*). The intracellular levels of TRF2 remained unchanged in the presence of 360A (2 μm), ruling out the possibility of decreased occupancy due to low TRF2 (Fig. 6*B*). Together, these suggested exclusion of TRF2 from G4 motifs in gene promoters by the G4-binding ligand 360A. Furthermore, we tested whether the presence of 360A affected the TRF2-G4 interaction *in vitro*. Association of recombinant TRF2 with G4 motifs (*SAMD14* and *CHRM2* promoters) was significantly impaired in the presence of 360A (Fig. S5).

We next asked whether the expression of the target genes was altered in presence of 360A. In five of the nine genes (*CHRM2*, *KCHN2*, *SAMD7*, *SAMD14*, and *THRA*), we found that TRF2-mediated suppression was rescued when cells were treated with 360A (Fig. 6*C*); however, on treatment with 360A alone, gene expression remained largely unchanged. CTCF was used as a negative control for mRNA expression as in earlier experiments.

## Discussion

Results herein not only show binding of TRF2 at thousands of extra-telomeric sites but also indicate that TRF2 associations are likely to modulate gene transcription at promoters harboring G4 motifs genome-wide. Validation of selected promoter sites based on ChIP-Seq findings, where TRF2 occupancy and TRF2-mediated expression of target genes was clearly observed, lends support to our conclusions. Finally, data showing the TRF2 as a DNA secondary structure G4 motif–binding factor and suggesting the role of TRF2-G4 interactions in transcription is noteworthy. Together, the results support intracellular formation of nonduplex G4 structures, including their role in gene regulation.

A multitude of TRF2 sites genome-wide overlap with regions that can adopt putative G4 motifs. This is of consequence from two interesting aspects. Although relatively recently noted, the role of G4 motifs in transcription regulation is being increasingly supported by the literature (31, 37, 55). Building on early studies reporting enrichment of sequence representing putative G4 motifs within promoters of a wide range of species (28, 31, 35, 57), several proteins have been found that engage G4 motifs (21, 37, 41, 58). Evidence supporting TRF2 as one such factor further substantiates this. Second, findings herein supporting the existence or formation of G4 motifs within live cells are of interest. Although specific antibodies have been shown to engage G4 motifs (44), questions regarding the natural or inherent presence of the G4 motifs remain (particularly because high-affinity antibody interactions may extrude formation of G4 motifs artificially). Global TRF2-G4 interactions indicated by ChIP-Seq studies, in addition to results from experiments with the ligand 360A supporting intracellular TRF2-G4 associations, reported here argue in favor of intrinsic G4 motifs that engage intracellular factors. However, further work will be required to fully understand this.

Previous work showed that TRF2 occupancy at gene promoters results in both activating and silencing histone marks (18, 47). However, among the genes that we studied, other than *OPN4*, TRF2 expression/occupancy resulted in repression of the target gene (Fig. 5*A*). Consistent with this, changes in the histone marks were also distinct for *OPN4* when compared with other promoters (Fig. 5*D*). Based on this, it is possible that TRF2 induces histone changes in a promoter-specific manner possibly in association with other co-activators/repressors. Other reports of TRF2 as a transcriptional regulator found that TRF2 activates *PDGFR*β (18) and acts as a repressor in case of *p21* (19). Together, these provide evidence for the bivalent nature of TRF2-mediated gene regulation.

We recently found that TRF2 recruits the RE1 silencing element (REST)-repressor complex to the *p21* promoter, resulting in repressive chromatin modifications and repression of *p21* (19). These findings suggest that mechanistically, in the case of genes undergoing TRF2-mediated repression, interaction with the REST-repressor complex may be involved. REST has been previously noted to facilitate genome-wide gene repression (59) and might require co-binders in a context-dependent fashion (60). Interaction of REST with TRF2 was also noted earlier in neuronal cells (61).

Notably, both the C-terminal MYB domain and the N-terminal basic domain of TRF2 were required for transcriptional regulation by TRF2. The TRF2-delM variant is known to function as a dominant negative mutant in double-strand telomere binding and telomere protection functions (10, 11). On the other hand, the TRF2 N-terminal B-domain was found to interact with the telomeric G4 motif in solution (21). In the case of TRF2-mediated transcription, overexpression of TRF2-delB or TRF2-delM mutants in the presence of endogenous TRF2 resulted in the same effect as TRF2 knockdown (Fig. 5*B*). However, this was not the case in cells lacking endogenous TRF2 (Fig. 5*C*). Taken together, it is possible that for promoters with G4 motifs, interaction of TRF2 with both the G4 motif and the flanking DNA is required and is through the B and M domains of TRF2.

Interestingly, unlike typical transcription factors, we were not able to identify a consensus TRF2-binding motif from our ChIP-Seq results. Previous reports of TRF2 extra-telomeric occupancy also failed to identify a consensus DNA sequence for TRF2 occupancy (15). This is not surprising because our results suggest that TRF2 binds to DNA structure instead of a sequence motif. Therefore, it is likely that the consensus TRF2 site represents a group of sequences that conform to the G4 motif. On the other hand, it is also possible that DNA binding by other TRF2-associated factors results in pulldown of multiple types of DNA sequences (*e.g.* TRF2-REST interactions noted earlier) (59, 60). From our ChIP-Seq data, it is evident that TRF2 binds a subset of all putative G4 motifs in the genome. It is therefore possible that the chromatin state of the region and/or occupancy of other regulatory DNA-binding proteins influences binding of TRF2 to G4 motifs in cells.

Recently, we observed the epigenetic state and expression of many TRF2 target promoters at sites remote from telomeres to be sensitive to telomere length (47). Except *CHRM2*, expression of all of the genes studied here was telomere-sensitive in either HT1080 or MRC5 cells (*KCHN2*, *OPN4*, and *SMAD7* expression was telomere-dependent in MRC5 cells only) (47). Furthermore, REST occupancy was also observed in *ANXA2*, *INHA*, *OBSL1*, *SAMD14*, and *THRA* promoters (47). Here, we find that several of these TRF2-binding sites are putative G4 motifs. It is therefore possible that extra-telomeric TRF2-G4 interactions are influenced by TRF2 association at the telomeres.

In conclusion, the findings herein collectively show the telomere-binding factor TRF2 as a likely global transcriptional regulator through promoter TRF2–G4 motif interactions. Based on this, future studies may help reveal mechanisms by which TRF2 modifies the transcriptional outcome of a gene promoter, resulting in possibly either up- or down-regulation of a gene, depending on the context. Perhaps, more importantly, such studies could help us to understand the roles of TRF2 both as a telomeric and an extra-telomeric factor.

## Experimental procedures

### Cell lines, media, and culture conditions

The HT1080 fibrosarcoma cell line was obtained from the NCCS (Pune, India). HT1080 cells were maintained in modified Eagle's medium supplemented with 10% fetal bovine serum. All cultures were grown in incubators maintained at 37 °C with 5% CO_2_.

### ChIP

ChIP assays were performed as per the protocol provided by Upstate Biotechnology with modifications as suggested in the Fast ChIP protocol. The antibody used for TRF2 ChIP (TRF2 Novus NB110-57130 has been reported earlier for immunoprecipitation of endogenous TRF2 (62, 63). Briefly, 4 million HT1080 cells were fixed with ∼1% formaldehyde for 10 min and lysed. Chromatin was sheared to an average size of ∼300–400 bp using Biorupter (Diagenode). 10% of the sonicated fraction was processed as input using phenol-chloroform and ethanol precipitation. ChIP was performed for endogenous/ectopically expressed protein using a 1:100 dilution (v/v) of the respective ChIP grade antibody incubated overnight at 4 °C. Immune complexes were collected using salmon sperm DNA-saturated magnetic Dyna-beads (50 μg/sample) and washed extensively. Phenol-chloroform-isoamylalcohol was used to extract DNA from the immunoprecipitated fraction. ChIP DNA was quantified by the Qubit HS ds DNA kit from Thermo Fisher Scientific. Quantified ChIP samples were validated by qRT-PCR. ChIP assays were performed using anti-TRF2 antibody (Novus Biologicals NB110-57130) and anti-rabbit IgG (Millipore) for isotype control in HT1080 cells. (For histone ChIP, the same general protocol was followed with relevant antibodies as follows: anti-H3, anti-H3K4me1, anti-H3K4me3, and anti-H3K27me3 (Abcam).) A previously reported negative control for TRF2 ChIP was used (47), CTCF (−ve control) sequence chr16:67594811–67594967, CCCCAAACTTATCTGGTCCCTTCACAGCAAAACCTCTCTCAAATTGCATACATGTGCTGTCTCCATTTCCTCACTTTCCTGGTGACTGTTTAACCCATTCCGGTCAGGTCCACCTCCCTGATATACTCACGTGAATCAAGCCAAGGCCATCAGTGA.

### Library preparation for ChIP-Seq

TRF2-bound or input DNA samples (prepared as described above) from HT1080 cells were quantified, and 20 ng from each sample was taken for end repair using the IlluminaTru-Seq sample preparation kit. Samples were purified using a PCR purification kit (Qiagen, Germany). Thereafter an “A” base was added to the samples' 3′-end using the Illumina sample preparation kit. After the end of the reaction, samples were again purified by a PCR purification kit (Qiagen). Then flow-cell primer-specific adapters were ligated to the ChIP DNA fragments, and samples were further purified by MinElute columns. Size selection was done after adapter ligation using 2% agarose gel. Gel extraction columns (Qiagen) were used to purify DNA fragments ranging between 150 and 350 bases. These eluted samples were then purified using MinElute columns and amplified for 18 cycles to enrich adapter-ligated DNA fragments. After PCR purification and elution, the DNA was quantified using the Picogreen method, and 7 pmol of each sample was sequenced on GAIIx (Illumina) according to the manufacturer's protocol.

### Analysis of high-throughput sequencing data

ChIP-Seq data were aligned to the human reference genome (hg19) using the Bowtie 2.1 short read aligner. Aligned reads were further de-duplicated using the samtools “rmdup” utility. Input DNA samples from HT1080 cells prepared as described above were sequenced along with TRF2-bound DNA samples. Reads generated from input were used to calculate background for ChIP-Seq peak identification processes. Peaks were called using MACS 1.4.2 (shift size, 125; *p* value cut-off, 1.00e−5; false discovery rate cut-off, 5%). Common peaks were identified as peaks whose coordinates intersected using BEDTool's intersect subcommand. For downstream analysis, common peaks were used (peak coordinates were overlapped so that the start coordinate is the minimum of replicate 1 and replicate 2 and the end coordinate is the maximum in replicate 1 and replicate 2. Raw sequencing data reads for ChIP-Seq are publicly available under the SRA study (accession number SRA 304653 (SRX1334027)). Refseq genes were mapped onto hg19 genome assembly using custom tracks of the UCSC genome browser to extract coordinates for TSS sites. For reanalysis, data from Simonet *et al.* (15) was obtained from GEO series accession number GSE26005: GSM638202 and GSM638203 for monoclonal and polyclonal ChIP, respectively. Peak calling was performed using identical parameters as used for our analysis using MACS 1.4.2. Fifty peak coordinates reported by Yang et al. (16) were used to find intersection with ChIP peaks (common from two replicates) reported by us.

### IDR analysis

The IDR analysis was performed as per ENCODE guidelines following the method reported by Bickel *et al.* (64). Publicly available IDR analysis code was downloaded (https://github.com/nboley/idr/archive/2.0.2.zip)^6^ and executed using Anaconda 5.1 Python distribution. The source file for the analysis was reformatted bed files of replicate1 and replicate2 of TRF2 ChIP-Seq peaks (see supporting information) to match the input file specifications of the IDR code.

### Circular dichroism

CD profiles (220–300 nm) were obtained for representative G4 motifs identified within 50 bp of the TSS of a gene and an overlap with TRF2 high-confidence ChIP seqpeaks. A list of oligonucleotides and respective mutated sequences is given in Fig. 3*A*. CD showed the formation of a G4 motif with the unaltered sequence, whereas a mutated G4 sequence gave partial/complete disruption of the G4 motif under similar conditions (buffer used for G-quadruplex formation: 10 mm sodium cacodylate and 100 mm KCl). The CD spectra were recorded on a Jasco-89 spectropolarimeter equipped with a Peltier temperature controller. Experiments were carried out using a 1-mm path-length cuvette over a wavelength range of 200–320 nm. Oligonucleotides were synthesized commercially by Sigma-Aldrich. 2.5 μm oligonucleotides were diluted in sodium cacodylate buffer (10 mm sodium cacodylate and 100 mm KCl, pH 7.4) and denatured by heating to 95 °C for 5 min and slowly cooled to 15 °C for several hours. The CD spectra reported here are representations of three averaged scans taken at 20 °C and are baseline-corrected for signal contributions due to the buffer.

### Dot blot assay

Input DNA and ChIP DNA (10 and 20 ng) were spotted on N+ Hybond membrane from Amersham Biosciences prewetted with 2× SSC (saline-sodium citrate) buffer and UV-cross-linked. The membranes were then blocked for 1 h at 37 °C using Rapid-Hyb buffer from Amersham Biosciences. Telomeric probes ((TTAGGG)_4_) or PCR-purified ALU probes were radiolabeled and hybridized to spotted DNA on the membranes at 37 °C overnight. The probes were washed off using successive 10-min washes of 2× SSC buffer, 2× SSC buffer with 0.1% SDS, and 0.2× SSC buffer. The membranes were then exposed to an imaging plate (Phosphor) and imaged using a Bio-Rad PhosphorImager.

### ELISA

Biotinylated oligonucleotides (Sigma-Aldrich) were used at a 5 μm concentration in 10 mm sodium cacodylate and 100 mm KCl buffer and denatured at 95 °C for 5 min, followed by slow cooling to room temperature to induce G-quadruplex formation. 384-well streptavidin-coated preblocked plates from Thermo Scientific (Pierce) were used for the ELISA. Biotinylated oligonucleotides were diluted to 5 pmol in 1× PBST buffer and loaded into each well. Oligonucleotides were incubated at 37 °C on a shaker for 2 h to allow streptavidin and biotin binding and then washed three times with 1× PBST buffer. Recombinant TRF2 protein was diluted in 1× PBST buffer and incubated with oligonucleotides for 2 h on a shaker at 4 °C and washed three times with 1× PBST buffer. In the competition assay, TRF2 was added along with competitor oligonucleotide. Anti-TRF2 antibody (Novus NB19-57130) was used in 1:1000 dilution (50 μl/well) and incubated for 1 h at room temperature on a shaker. Wells were washed three times with 1× PBST. Alkaline phosphatase–conjugated anti-IgG antibody (Sigma) was used in a 1:1000 dilution (50 μl/well) and incubated for 45 min at room temperature on a shaker, and then wells were washed once with 1× PBST and twice with 1× PBS. 10 μl of BCIP/NBT substrate was added to each well, and absorbance was recorded at 610-nm wavelength for 1 h with a 10-min interval on a TECAN multimode reader. Two controls were used in ELISA to subtract background binding of antibody and protein: 1) for the protein-negative control, except for TRF2 protein, all other reagents were added to determine the background binding of antibodies, and 2) for the oligonucleotide-negative control, except for oligonucleotide, all other reagents along with increasing concentrations of protein were added to determine background binding of the protein. The absorbance obtained from control wells was subtracted from absorbance obtained from experiment wells to get specific binding. GraphPad Prism version 7 software was used for analysis.

### Vector constructs

The bacterial expression vector, pTRC-hisTRF2, was received as a gift from Dr Giraud Panis (CNRS, France). The pCMV6-myc-DDK(FLAG)-TRF2 vector was procured from Origene (RC223601). The mutant delB and delM constructs used in the study have been reported previously (19). TRF2 shRNA was procured from Origene (TL308880).

### Protein purification

TRF2 WT was purified using *Escherichia coli* Rosetta Gami2 cells. In brief, transformed cells were inoculated into a 5-ml culture with ampicillin antibiotic and kept at 37 °C overnight in a shaker incubator. The next day, 1 ml of culture was inoculated in 500 ml of fresh Luria–Bertani broth with antibiotic and allowed to grow until optical density reached 0.8 at 600 nm. Then culture was induced with a 0.1 mm final concentration of isopropyl 1-thio-β-d-galactopyranoside and kept overnight in a shaker incubator at 18 °C. The next day, the culture was pelleted down and sonicated in lysis buffer. 200 μl of His-pure nickel-nitrilotriacetic acid beads (Thermo Scientific) were added and incubated at 4 °C in a rotatory shaker. Beads were washed with a 20-ml solution of 20, 30, 40, and 60 mm imidazole. Protein was eluted with 4 ml of 250 mm imidazole solution. The concentration of purified protein and buffer exchange to remove imidazole were done by using Millipore 4-ml concentrator columns. Purified protein was quantified by the BCA method (Thermo Scientific BCA kit).

### G4-FID assay

Experiments were conducted for the FID assay as reported earlier (56). Briefly, PG4 oligonucleotides were dissolved in 100 mm KCl^+^, 10 mm sodium cacodylate buffer, heated up to 95 °C for 5 min, and allowed to cool slowly to room temperature. The quadruplex structure formation was confirmed by CD spectral analysis. DNA was added to a final concentration of 0.25 μm along with 2 molar eq of the fluorescent intercalator ligand TO and mixed well, and the fluorescence spectra at *t* = 0 and *t* = 5 min over a range of 490–750 nm was observed. Increasing concentrations of recombinant TRF2 (0–10 molar eq) were added to displace TO from the DNA by successive additions of small volumes of buffer containing TRF2, and the fluorescence spectra were recorded after each addition. Percentage TO displacement was calculated by the following formula,

(Eq. 1) $${\text{TOD}}_{\text{x}}=100-\left(\left(\text{FAx}-\text{FA}1\right)\times 100\right)$$

where FA1 represents the fluorescence area of the spectrum recorded after the addition of TO, and FA*x* is the fluorescence area of the spectrum recorded after the *x*th addition of the ligand.

Percentage TO displacement was plotted as a function of TRF2 concentration. The amount of TRF2 required for displacement of 50% TO was calculated as DC_50_; TRF2 concentration *versus* percentage TO displacement was used for estimating indicative *K_d_* of TRF2-G4 interaction using GraphPad.

### Transfections

TRF2 WT (Myc/DDK tag) or mutant mammalian expression vector pCMV6 was transfected into HT1080 cells that were 60% confluent using Lipofectamine 2000 transfection reagent (following the manufacturers' protocol). 2–4 μg of plasmid was used for transfection in a 35-mm well for each case.

In the case of TRF2 shRNA (Origene), 4 μg of plasmid was used for transfection in a 35-mm well for each case, and Fugene HD transfection reagent was used as per the manufacturer's protocol. Cells were then maintained in 1 μg/ml puromycin for 72 h before transfection of TRF2 constructs.

### Real-time PCR

Total RNA was isolated using TRIzol® reagent (Invitrogen, Life Technologies, Inc.) according to the manufacturer's instructions. A relative transcript expression level for genes was measured by quantitative real-time PCR using a SYBR Green–based method. Average -fold changes were calculated by the difference in threshold cycles (*Ct*) between test and control samples. The *GAPDH* gene was used as an internal control for normalizing the cDNA concentration of each sample.

### Western blotting

For Western blot analysis, protein lysates were prepared by suspending cell pellets in 1× cell culture lysis buffer (Promega). Protein was separated using 10% SDS-PAGE and transferred to polyvinylidene difluoride membranes (Immobilon FL, Millipore Corp.). After blocking, the membrane was incubated with primary antibodies. Postincubation with primary antibodies, the membrane was washed with 1× PBS and then incubated with appropriate secondary antibodies. Following secondary antibody incubation, the membrane was washed with 1× PBS. The blot was finally developed using HRP substrate and reagents from Millipore. Primary antibodies used were TRF2 (Novus, NB110-57130), DDK (Sigma, F1804), and GAPDH (Santa Cruz Biotechnology, Inc., G-9). Secondary antibodies used were mouse HRP (Cell Signaling Technology) and rabbit HRP (Cell Signaling Technology).

All primary antibodies were used in 1:1000 dilution. Secondary antibodies used were mouse HRP (Cell Signaling Technology) and rabbit HRP (Cell Signaling Technology). All secondary antibodies were used in 1:3000 dilution.

### Antibodies used

For chromatin immunoprecipitation, anti-TRF2 antibody (Novus Biologicals, NB110-57130), anti-H3 (ab1791, Abcam), anti-H3K4me1 (ab8895, Abcam), anti-H3K4me3 (ab8580, Abcam), anti-H3K27me3 (ab192985, Abcam), and anti-rabbit IgG/anti-mouse IgG (Millipore) were used. For ELISA, anti-TRF2 antibody (Novus Biologicals NB110-57130) was used. For Western blotting, the following primary antibodies were used: TRF2 (Novus, NB110-57130), DDK (Sigma, F1804), and GAPDH (Santa Cruz Biotechnology, G-9).

### Dilutions

The following dilutions were used: ChIP, 1:100 (v/v); ELISA, 1:1000 (v/v); Western blotting, 1:1000 (v/v) for primary antibodies and 1:3000 (v/v) for secondary antibodies.

## Author contributions

A. K. M., S. S., and S. C. conceptualization; A. K. M., S. S., S. B., D. S., A. K., D. D., and S. C. resources; A. K. M., S. S., S. B., and S. C. data curation; A. K. M. and S. S. software; A. K. M., S. S., S. B., R. K., P. K., A. H., P. S., and S. C. formal analysis; A. K. M., S. S., S. B., and A. H. validation; A. K. M., S. S., and S. C. investigation; A. K. M., S. S., R. K., P. K., D. D., and S. C. visualization; A. K. M., S. S., S. B., D. S., A. K., D. D., and S. C. methodology; A. K. M., S. S., and S. C. writing-original draft; S. B. writing-review and editing; D. D. and S. C. supervision; S. C. funding acquisition.

## Supplementary Material

Supporting Information

## Footnotes

6 Please note that the JBC is not responsible for the long-term archiving and maintenance of this site or any other third party hosted site.

## Footnotes

5 The abbreviations used are:

ATM: ataxia-telangiesctasia mutated
ATR: ATM and RAD3-related
G4: G-quadruplex
PG4: potential G4
TSS: transcription start site
IDR: irreproducible discovery rate
FID: fluorescent intercalator displacement
TO: thymidine orange
H3K4me1: histone H3 Lys-4 monomethylation
H3K4me3: histone H3 Lys-4 trimethylation
H3K27me3: histone H3 Lys-27 trimethylation
REST: RE1 silencing element
qRT-PCR: quantitative RT-PCR
HRP: horseradish peroxidase
GAPDH: glyceraldehyde-3-phosphate dehydrogenase.
