# Supplementary material for: Telomere repeat–binding factor 2 binds extensively to extra-telomeric G-quadruplexes and regulates the epigenetic status of several gene promoters
Source: J Biol Chem. 2019 Oct 1;294(47):17709–22. doi: 10.1074/jbc.RA119.008687 (PMC6879327; doi:10.1074/jbc.RA119.008687)
Supplement: Supporting Information [file supp_294_47_17709__index.html]

Telomere repeat–binding factor 2 binds extensively to extra-telomeric G-quadruplexes and regulates the epigenetic status of several gene promoters — TRF2 binds G-quadruplexes genome-wide — Telomere repeat–binding factor 2 binds extensively to extra-telomeric G-quadruplexes and regulates the epigenetic status of several gene promoters — EDITORS' PICK: TRF2 binds to promoter G-quadruplexes genome-wide — Supporting Information 

# Telomere repeat–binding factor 2 binds extensively to extra-telomeric G-quadruplexes and regulates the epigenetic status of several gene promoters

## Supporting Information

- excel file with Supporting Information - Details of the ChIP seq peaks, IDR analysis and reanalyis of previoulsy published ChIP seq
- Supporting Information (to be published online) - SUPPLEMENTARY FIGURES
