# Supplementary material for: Telomere repeat–binding factor 2 binds extensively to extra-telomeric G-quadruplexes and regulates the epigenetic status of several gene promoters
Source: J Biol Chem. 2019 Oct 1;294(47):17709–22. doi: 10.1074/jbc.RA119.008687 (PMC6879327; doi:10.1074/jbc.RA119.008687)
Supplement: Supporting Information [file supp_RA119.008687_144640_2_supp_399055_pytt68.pdf]

Supplementary Figure 1

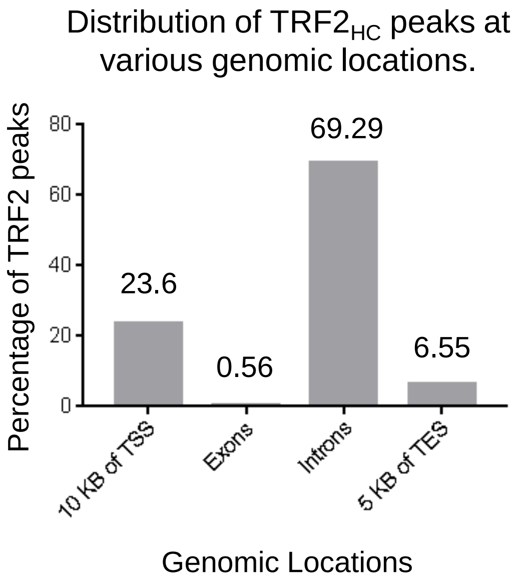

Supplementary Figure 2

(A)

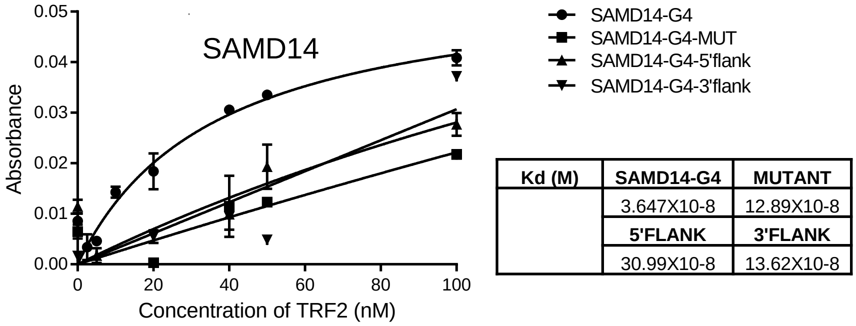

Kd (M)	SAMD14-G4	MUTANT
	3.647X10-8	12.89X10-8
	5'FLANK	3'FLANK
	30.99X10-8	13.62X10-8

Binding affinity of recombinant TRF2 to promoter G4 sequences determined using ELISA with biotin labelled oligos. 3' biotin tagged *SAMD14* and *CHRM2* WT sequences (along with G4 mutant sequences or 5' and 3' flanking sequences of same length, as negative controls) were used for the assay.

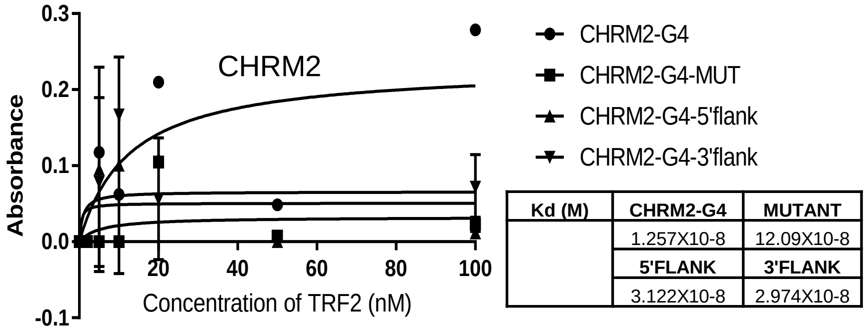

Kd (M)	CHRM2-G4	MUTANT
	1.257X10-8	12.09X10-8
	5'FLANK	3'FLANK
	3.122X10-8	2.974X10-8

(B)

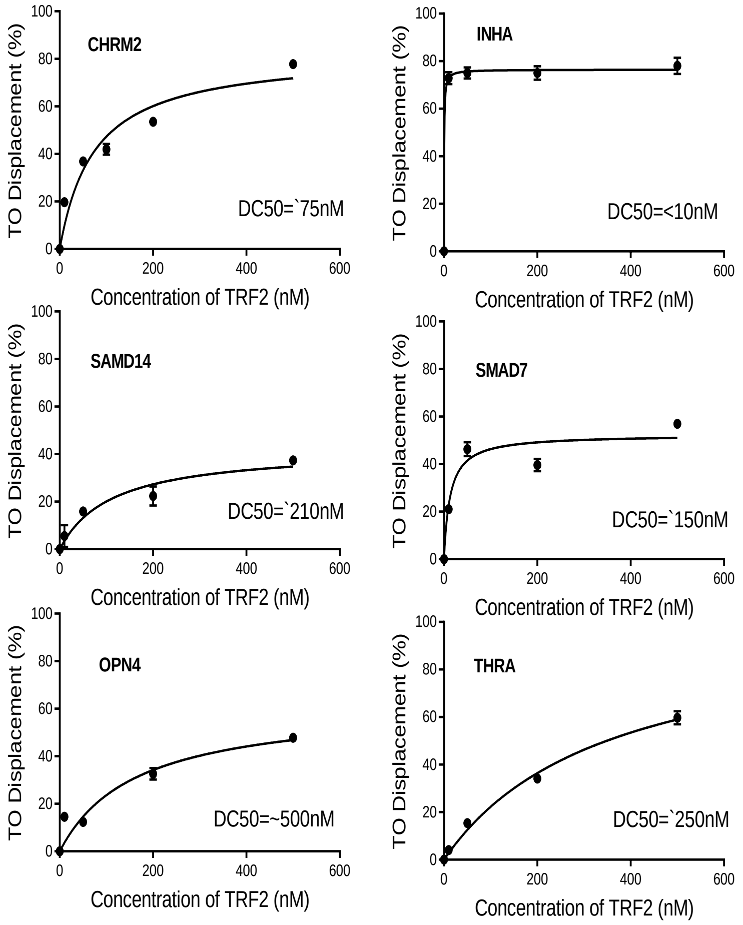

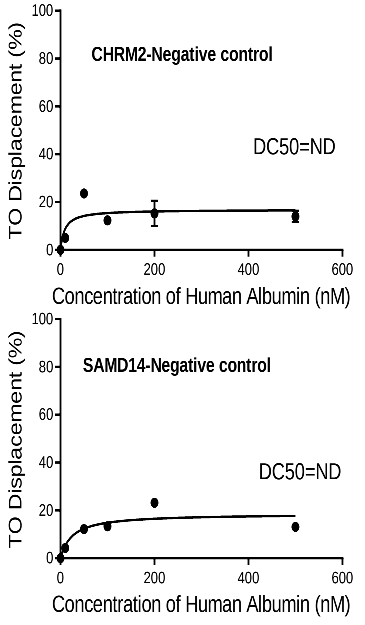

Association of purified TRF2 protein was checked with G4 motifs through displacement of the G4-bound fluorescent dye Thiazole Orange (TO) by TRF2. The assay was performed for six representative G4 forming sequences. DC_{50} values were calculated for each as mentioned in Methods. Human albumin instead of TRF2 was used as negative control for two representative cases; ND: not determined.

Supplementary Figure 3

(A)

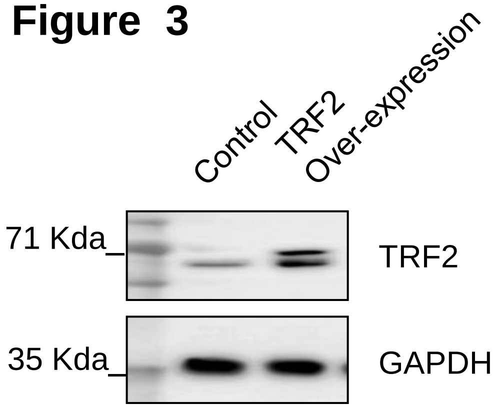

TRF2 level was checked by western blot in untransfected and TRF2 transient over- expression conditions.GAPDH was used as loading control

(B)

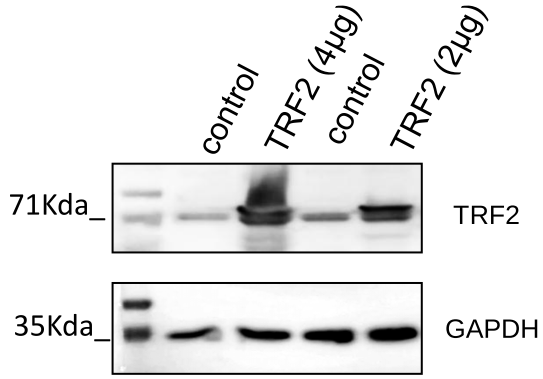

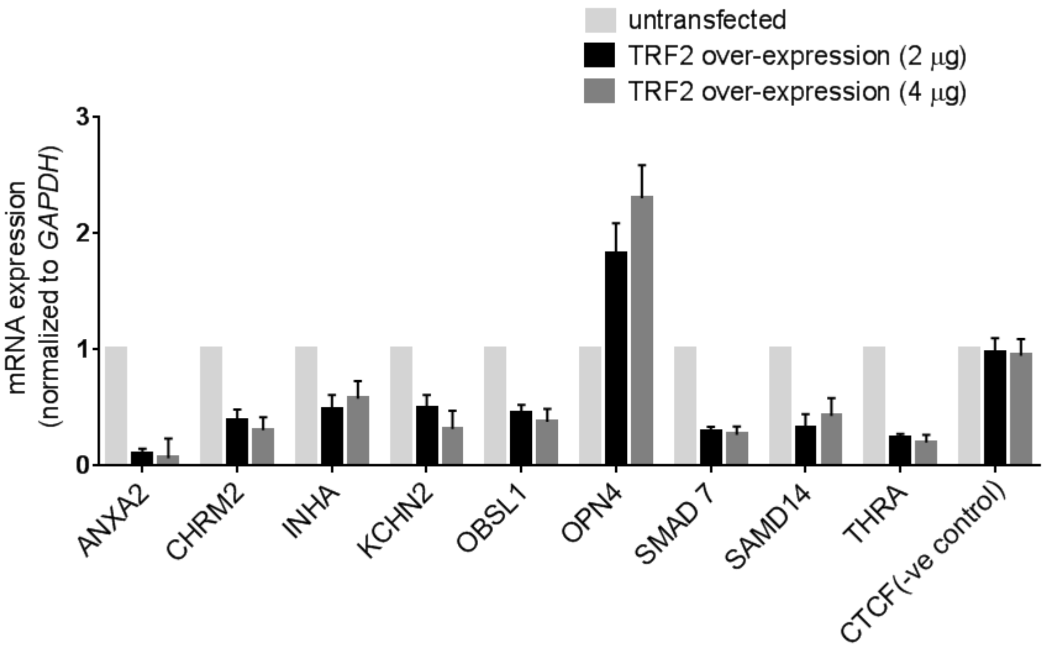

Gene expression of target genes measured by qRT-PCR in untransfected and TRF2 over-expressed (2µg and 4µg) conditions in HT1080 cells. *GAPDH* expression used for normalization; error bars correspond to ± SD from two independent experiments.

Supplementary Figure 4

(A)

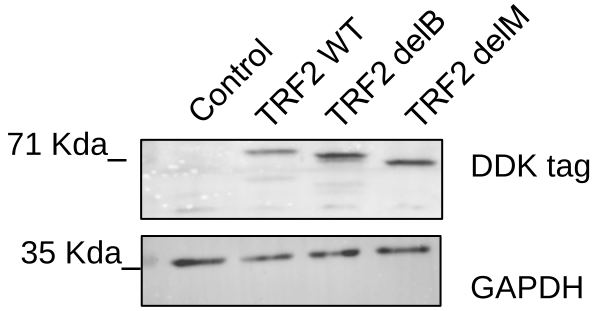

Transient over-expression of TRF2 WT, delB and delM mutants in HT1080 cells was confirmed by probing for DDK tag. GAPDH was used as loading control

(B)

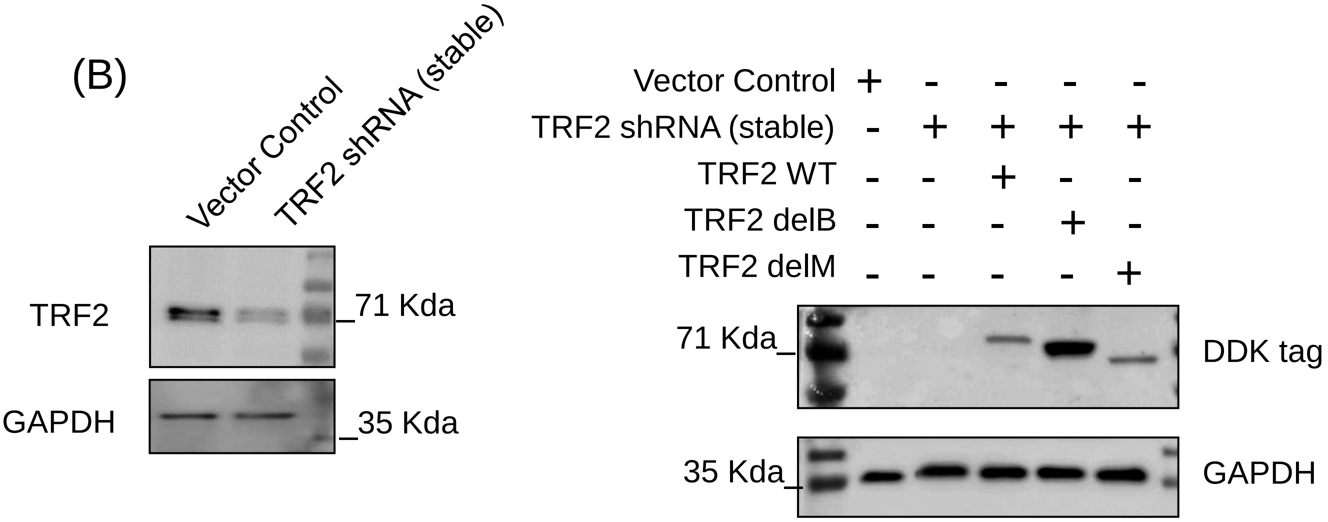

Stable silencing of TRF2 was achieved by transfection of TRF2 shRNA followed by puromycin selection. Stable silencing was confirmed by Western blot. Transient over-expression of DDK tagged TRF2 WT, delB and delM mutants in HT1080 cells with stable TRF2 silencing was confirmed by probing for DDK tag. GAPDH was used as loading control

Supplementary Figure 5

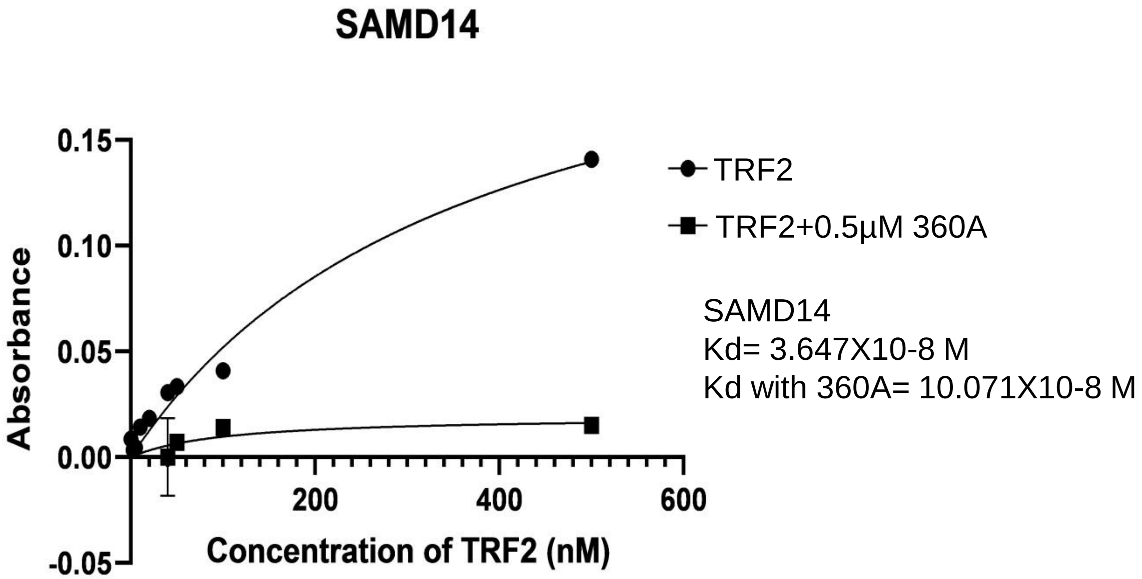

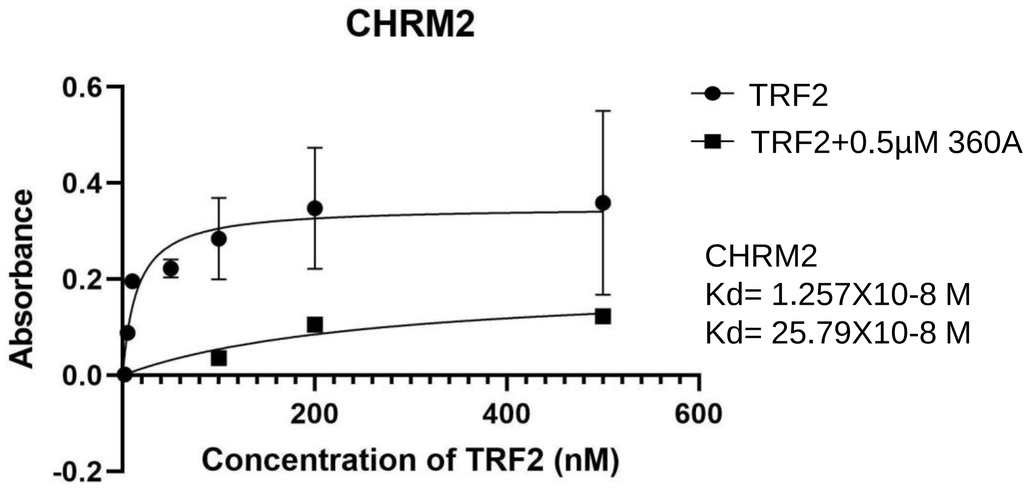

Binding affinity of recombinant TRF2 to promoter G4 sequences determined using ELISA with biotin labelled oligos. 3’ biotin tagged *SAMD14* and *CHRM2* WT sequences in presence and absence of ligand 360A
